# Toward Elucidating Epigenetic and Metabolic Regulation of Stem Cell Lineage Plasticity in Skin Aging

**DOI:** 10.3389/fcell.2022.903904

**Published:** 2022-05-19

**Authors:** Ying Lyu, Yejing Ge

**Affiliations:** Department of Cancer Biology, The University of Texas MD Anderson Cancer Center, Houston, TX, United States

**Keywords:** skin aging, stem cell lineage plasticity, inflammaging, epigenetics, metabolism

## Abstract

Skin is the largest organ in human body, harboring a plethora of cell types and serving as the organismal barrier. Skin aging such as wrinkling and hair graying is graphically pronounced, and the molecular mechanisms behind these phenotypic manifestations are beginning to unfold. As in many other organs and tissues, epigenetic and metabolic deregulations have emerged as key aging drivers. Particularly in the context of the skin epithelium, the epigenome and metabolome coordinately shape lineage plasticity and orchestrate stem cell function during aging. Our review discusses recent studies that proposed molecular mechanisms that drive the degeneration of hair follicles, a major appendage of the skin. By focusing on skin while comparing it to model organisms and adult stem cells of other tissues, we summarize literature on genotoxic stress, nutritional sensing, metabolic rewiring, mitochondrial activity, and epigenetic regulations of stem cell plasticity. Finally, we speculate about the rejuvenation potential of rate-limiting upstream signals during aging and the dominant role of the tissue microenvironment in dictating aged epithelial stem cell function.

## Introduction

As the body’s largest organ, skin harbors a cadre of cell types. First and foremost, epithelial cells serve as the fundamental units of our barrier; they reside in the interfollicular epidermis and pilosebaceous unit, the latter including the sebaceous gland and the hair follicle (Hardy, 1992; Fuchs, 1995; Paus and Cotsarelis, 1999; Watt, 2001; Lopez-Pajares et al., 2013). Fibroblasts in the dermis secrete bulk extracellular matrix in the tissue for not only structural support but also mediate signaling (Gurtner et al., 2008; Sennett and Rendl, 2012; Heitman et al., 2019; Plikus et al., 2021; McAndrews et al., 2022). Subcutaneous adipocytes and dermal pre-adipocytes exhibit remarkable lineage plasticity to mediate metabolic and signaling regulations (Horsley and Watt, 2017). Immune cells including those of the innate and adaptive systems provide tissue surveillance (Allison and Havran, 1991; Merad et al., 2008; Heath and Carbone, 2013), support repair and regeneration (DeStefano and Christiano, 2014; Ali et al., 2017; Eming et al., 2017), and communicate with the microbiome (Nakatsuji and Gallo, 2012; Byrd et al., 2018; Kobayashi et al., 2019). Oxygen and nutrients are exchanged via the endothelial and lymphatic vasculature, sensations are conducted through the intertwining neuronal network, ultraviolet radiation protection is afforded by melanocytes, and hair follicles are erected by arrector pili muscles; all of these cell types exert their respective functions while maintaining crosstalk with juxtaposed epithelial stem cells (Nishimura et al., 2005; Brownell et al., 2011; Fujiwara et al., 2011; Chang et al., 2013; Gur-Cohen et al., 2019; Shwartz et al., 2020; Zhang et al., 2020).

**FIGURE 1:**
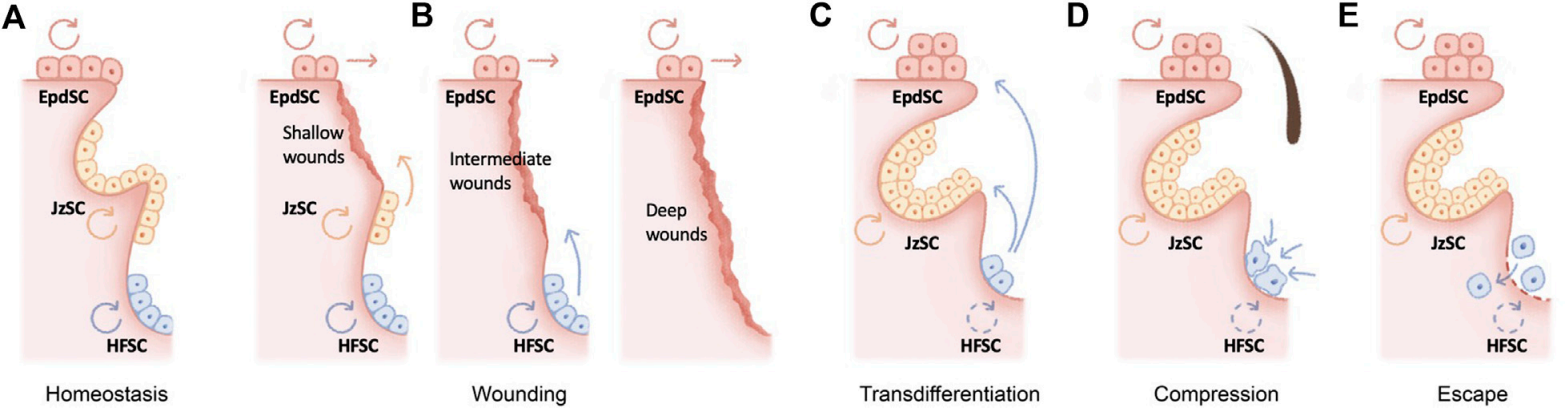
Molecular mechanisms underlying hair follicle miniaturization in aging **(A)** The hair follicle as a major skin appendage is maintained by hair follicle stem cells (HFSCs) residing in the bulge, an anatomic location beneath the sebaceous gland and isthmus of the pilosebaceous unit. HFSCs fuel the cyclic regeneration of hair follicles during homeostatic hair growth, whereas epidermal stem cells (EpdSC) and junctional zone stem cells (JzSCs) maintain the epidermis and junctional zone, respectively **(B)** HFSCs drive both follicular and epidermal regeneration during wound repair. Depending on the wounding depth, different populations of stem cells are induced to contribute to wound repair **(C–E)** Several cellular and molecular mechanisms have been recently proposed to explain the fate of aging HFSCs in murine models, including epidermal transdifferentiation **(C)**, mechanical compression *in situ* **(D)**, and escape into dermis **(E)**.

The skin epithelium is maintained by its resident stem cells, harboring the capacity for long-term self-renewal and multi-lineage differentiation. The hair follicle as a major skin appendage is maintained by hair follicle stem cells (HFSCs) residing in the bulge, an anatomic location beneath the sebaceous gland and isthmus of the pilosebaceous unit (Cotsarelis et al., 1990) (Figure 1A). HFSCs fuel the cyclic regeneration of hair follicles during homeostatic hair growth (Morris et al., 2004; Tumbar et al., 2004; Greco et al., 2009; Hsu et al., 2011). They contribute to both follicular and epidermal regeneration during transplantation and wound repair (Taylor et al., 2000; Blanpain et al., 2004; Claudinot et al., 2005; Ito et al., 2005; Levy et al., 2005), and they initiate skin squamous cell carcinomas upon oncogenic transformation (Lapouge et al., 2011). Likewise, exhibiting remarkable plasticity, several HFSC populations adjacent to the bulge contribute to wound repair and tumorigenesis (Nijhof et al., 2006; Jaks et al., 2008; Jensen et al., 2009; Snippert et al., 2010; Page et al., 2013; Donati et al., 2017; Ge et al., 2017; Ge and Fuchs, 2018) (Figure 1B).

Aging in the skin is graphically pronounced and includes a wrinkly surface due to dermal extracellular matrix atrophy and gray hair owing to a loss of melanocytes. Accompanying these overtly notable signs, the old skin manifests many hallmarks of aging (Lopez-Otin et al., 2013) such as stem cell exhaustion, genotoxic stress, metabolic deregulation, and epigenetic erosion. In our current essay, we focus on skin aging, especially in the context of stem cell function and the origin and consequence of lineage deregulation. Whenever applicable, we compare mammalian skin to model organisms and adult stem cell of other tissues and organs. Overall, we entertain the idea that skin is an ideal system to understand and tackle organismal aging.

## Molecular Mechanisms Underlying Hair Follicle Miniaturization

Stem cells decline over time in number and/or in activity across organs and tissues. Curiously, this functional decline is often accompanied by skewed lineage output. For example, aging in the hematopoietic system is signified by pronounced myeloid lineage expansion at the expense of lymphoid cells (Sudo et al., 2000) (Cho et al., 2008; Florian et al., 2013). Aged skeletal stem cells have decreased bone- and cartilage-forming potential but produce more stromal lineages, leading to not only bone fragility but also hematopoietic skewing (Ambrosi et al., 2021). Secretory cell types dominate the aged intestinal stem cell output (Nalapareddy et al., 2017). The question of the fate of HFSCs during aging is a particularly intriguing one. Consistent with the lineage-skewing phenomenon at the organ level, hair follicles undergo miniaturization as HFSCs are diminished during aging, resulting in macroscopically sparse hair. In contrast, the epidermis and sebaceous gland undergo hyperplasia. Several cellular and molecular mechanisms have been recently proposed to explain the fate of aging HFSCs in murine models (Figures 1C–E).

In the skin, stem cell lineage infidelity where HFSCs expand their fate to regenerate the epidermis is observed during wound repair (Ge et al., 2017) (Figure 1B) and could be recapitulated upon the ablation of several key HFSC fate transcription factors in adult skin, including SOX9 (Kadaja et al., 2014) and NFIB/NFIX (Adam et al., 2020), suggesting a default HFSC response of epidermal differentiation under injury. In a more extreme scenario, epidermal cysts form in Notch signaling–deficient skin, suggesting blocked hair follicle differentiation (Yamamoto et al., 2003; Pan et al., 2004; Vauclair et al., 2005; Estrach et al., 2006; Demehri and Kopan, 2009). During aging, stress signals including DNA damage and a high-fat diet have been shown to drive epidermal conversion of HFSCs by compromising basement membrane integrity (Matsumura et al., 2016), altering stem cell symmetric divisions (Matsumura and Nishimura, 2021), and inducing oxidative stress (Morinaga et al., 2021), all of which contribute to the epidermal conversion of HFSCs and hair follicle miniaturization (Figure 1C).

On the other hand, deficiency of several HFSC quiescence regulators, such as LHX2 (Folgueras et al., 2013) or beta-catenin (Lien et al., 2014), appears to push HFSCs into the sebaceous gland lineage. In this regard, loss of BMP (Guha et al., 2004; Plikus et al., 2004; Qiu et al., 2011) or aberrant activation of LEF1 (Merrill et al., 2001; Niemann et al., 2002; Petersson et al., 2011), NOTCH1 (Estrach et al., 2006), and GLI2 (Allen et al., 2003; Gu and Coulombe, 2008) also manifests as ectopic sebaceous glands, although in some of these scenarios, it remains unclear whether sebaceous glands were mis-specified from HFSCs at the expense of hair follicles. Since sebaceous gland hyperplasia, like that of the epidermis, is a prominent signature of skin aging, it would be tempting to speculate that aged HFSCs also skew toward sebaceous gland differentiation, resulting in hair follicle miniaturization (Figures 1B,C).

An alternative model for the loss of aged HFSCs was recently proposed (Figure 1D): mechanical compression induces Piezo channel activation, calcium influx, and subsequently HFSC apoptosis (Xie et al., 2022). While the loss of inhibitory K6+ niche cells is known to cause HFSC activation (Hsu et al., 2011), in this context, mechanical cues play a dominant function, since the reinsertion of the hair shaft alone without the K6+ cells returned bulge HFSCs to quiescence (Xie et al., 2022). Corroborating this model, repetitive hair depilation induces hair follicle aging (Keyes et al., 2013; Lay et al., 2016), likely evoking mechanical compressions and the Piezo1-calcium-TNFα axis (Xie et al., 2022). Therefore, mechanical compressions induced HFSC apoptosis is likely a major contribution of HFSC exhaustion and hair follicle miniaturization during aging.

Remarkably, *via* intravital imaging, aged HFSCs have been shown to escape into the dermis (Zhang et al., 2021) (Figure 1E). This escape could be recapitulated by depletion of HFSC quiescence regulators FOXC1 (Lay et al., 2016; Wang et al., 2016) and NFATC1 (Keyes et al., 2013) in the hair follicles and is caused by the aging-associated loss of extracellular matrix proteins (Ge et al., 2020; Zhang et al., 2021). On the other hand, deregulation of the aging extracellular matrix due to niche stiffening underlies nuclear cytoskeletal remodeling and subsequently epigenome remodeling in the aging HFSCs (Koester et al., 2021), providing an important molecular link as to how the extracellular matrix niche determines aged HFSC fate (Ge et al., 2020; Koester et al., 2021).

## Impact of Genotoxic Stress on Skin Aging

**FIGURE 2:**
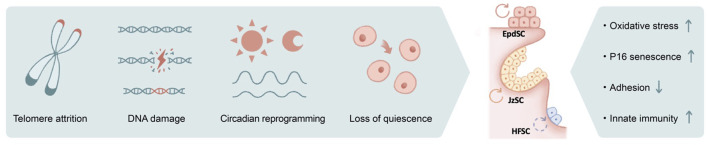
The impact of genotoxic stress on skin aging. Numerous causes of genotoxic stresses including telomere attrition, DNA damage, circadian reprograming, and loss of quiescence could lead to stem cell functional decline in aging. The outcomes include increased oxidative stress, induction of P16 and cellular senescence, loss of cell adhesion, and activated innate immune pathways, among others.

Telomeres shorten each time the genome duplicates, serving as a major driver for replicative aging. In mammals, telomerase expression is present and often abundant in adult stem cells (Sharpless and DePinho, 2004), including those of the skin (Figure 2). Among the defects in many highly proliferative organs, a signature phenotype of telomerase-deficient mice is alopecia and hair graying (Lee et al., 1998; Rudolph et al., 1999; Flores et al., 2005; Sarin et al., 2005). Likewise, in humans, mutations in the gene encoding for dyskerin, critical for telomere stability, lead to dyskeratosis congenita, a progressive bone-marrow failure syndrome characterized by abnormal skin pigmentation (Mitchell et al., 1999; Vulliamy et al., 2001).

Similar to telomere attrition, DNA damage–induced genotoxic stress due to replication error, mutagens, and reactive oxygen species (ROS) is well documented to accelerate aging (Figure 2). Among DNA repair pathways, it has been shown that non-homologous end joining (NHEJ) is specifically enhanced in quiescent hematopoietic stem cells (HSCs), whereas committed progenitors preferentially use homologous recombination as their repair mechanism (Mohrin et al., 2010). Similarly, quiescent bulge HFSCs are relatively protected against ionizing radiation, likely because of their elevated anti-apoptotic programs and enhanced NHEJ capacity (Sotiropoulou et al., 2010). Deficiencies in various NHEJ components (Difilippantonio et al., 2000; Gao et al., 2000) (Baker et al., 2004; Nijnik et al., 2007), spindle assembly checkpoint proteins (Baker et al., 2004), and DNA damage response pathways (Ito et al., 2004; Ruzankina et al., 2007) collectively contribute to premature aging of the hair follicles.

Disruption of the circadian clock results in pre-mature aging (Janich et al., 2011; Kondratov et al., 2006; Yu et al., 2021), and its associated molecular mechanisms are only beginning to emerge (Figure 2). It has been suggested the transit-amplifying cells of the hair follicles exhibit elevated sensitivity toward DNA damage during the day when their mitotic activities peak (Plikus et al., 2013), whereas the epidermis preferentially proliferates at night to avoid the high level of oxidative phosphorylation (with its byproduct ROS) during the day, presumably as a protective mechanism against genotoxicity (Stringari et al., 2015). In aged epidermal stem cells, arrhythmic and prolonged DNA replication combined with otherwise-normal oscillatory oxidative phosphorylation programs may therefore underlie the increased oxidative damage (Solanas et al., 2017). Nevertheless, complete disruption of the clock did not recapitulate selective rhythmic deregulation in a physiological aging setting, nor did circadian rewiring induced by a high-fat diet, suggesting the direct cause of aging-associated circadian reprogramming remains unclear (Solanas et al., 2017).

Furthermore, chronic inflammatory signals also contribute to DNA damage and genotoxicity. For example, IFNα stimulate HSCs to exit quiescence via IFNAR and STAT1 signaling, elevate mitochondrial ROS levels, and lead to DNA damage (Essers et al., 2009; Sato et al., 2009; Walter et al., 2015) (Figure 2). ROS may act upstream of DNA damage, evidenced by the observation that antioxidant treatment is able to suppress DNA damage and thus delay HSC aging (Essers et al., 2009; Walter et al., 2015). Genotoxic stress could further exacerbate ROS deregulation and subsequently activate p38MAPK and upregulate the expression of P16, resulting in HSC exhaustion (Ito et al., 2004) (Ito et al., 2006). In the case of HFSCs, DNA damage induces proteolysis of collagen XVII, a critical cell junction collagen, and induces stem cell differentiation (Matsumura et al., 2016). Likewise, ionizing radiation–induced DNA damage leads to melanocyte stem cell exhaustion and hair graying (Inomata et al., 2009). Strikingly, hair graying is completely suppressed in mice that are deficient in the double-stranded DNA-sensing cGAS/STING pathway (Dou et al., 2017), suggesting that the innate immunity pathway serves as a key mediator of radiation-induced aging.

## Nutritional and Metabolic Regulation of Skin Aging

**FIGURE 3:**
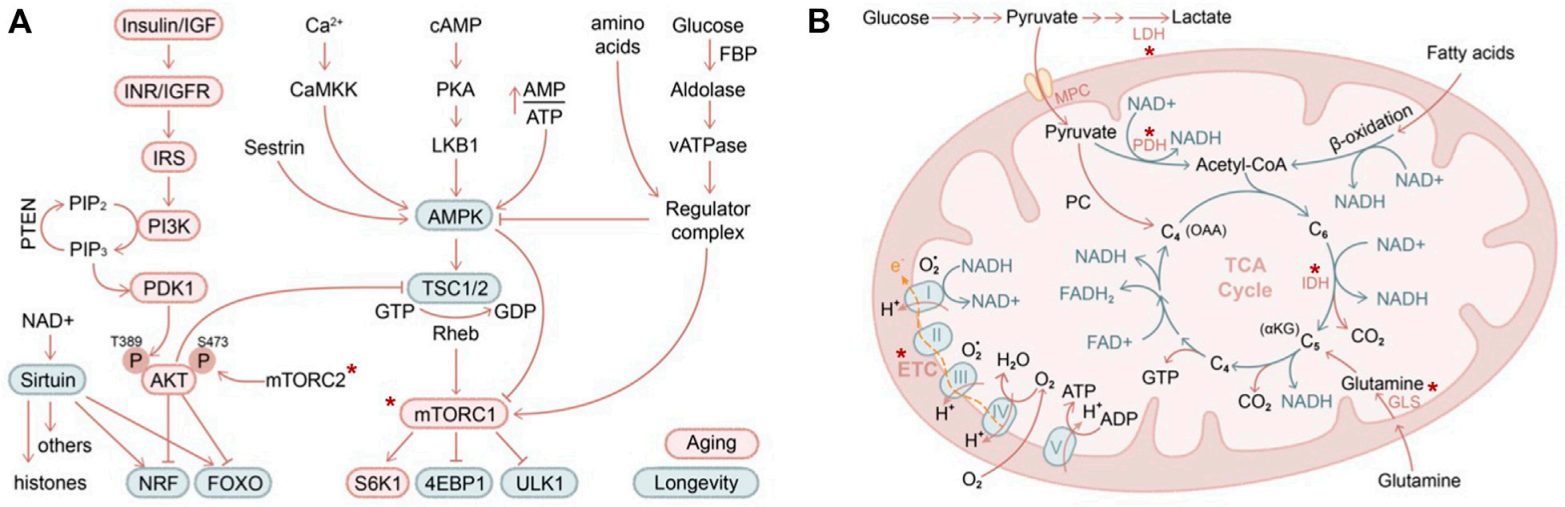
Nutritional and metabolic regulation of skin aging **(A)** IIS (insulin/insulin-like growth factor) and AMPK/mTOR are two major pathways in regulating nutrients and metabolism that have been described in contributing to organismal aging (red circle) and longevity (green circle). Asterix indicates components of these pathways that have been demonstrated to contribute to skin aging **(B)** Mitochondrial biology is closely intertwined with aging biology. Highlighted in red are several key genes that have been shown to regulate skin stem cell aging *in vivo*. LDH, lactate dehydrogenase; PDH, pyruvate dehydrogenase; MPC, mitochondrial pyruvate carrier; GLS, glutaminase; IDH, isocitrate dehydrogenase (converting isocitrate into alpha-ketoglutarate). Asterix indicates components of these pathways that have been demonstrated to contribute to skin aging.

Pioneering work has established several nutrient-sensing and energy-sensing pathways to be critically involved in lifespan extension across model organisms (Figure 3A), including insulin/insulin-like growth factor (IIS) (Kenyon, 2010), AMP-activated protein kinase (AMPK) (Herzig and Shaw, 2018), mammalian or mechanistic target of rapamycin (mTOR) (Wullschleger et al., 2006; Laplante and Sabatini, 2012), and sirtuin (Haigis and Guarente, 2006; Longo and Kennedy, 2006; Houtkooper et al., 2012) pathways. While mechanistic studies of these pathways in skin aging are rapidly emerging, in this section, we will review the historical aspects of these molecular mechanisms in various model organisms and adult stem cells in order to provide a broader context for skin aging.

In the IIS pathway, the selective ablation of signaling cascade components led to activation of downstream transcription factors FOXO (Lee et al., 2003; Murphy et al., 2003) and NRF (Sykiotis and Bohmann, 2008; Tullet et al., 2008), eliciting cellular protective programs against oxidative stress and promoting longevity (Tothova et al., 2007; Blackwell et al., 2015). AMPK senses cellular energy decline and activates a plethora of catabolic pathways while suppressing anabolic processes, promoting lifespan extension (Mair et al., 2011; Herzig and Shaw, 2018). The antidiabetic antineoplastic drug metformin exerts both AMPK-dependent (Ma et al., 2022) and AMPK-independent activities (Foretz et al., 2014) and, when used at a high dose, inhibits mitochondrial electron transport chain complex I (described further below), all of which may contribute to its utility in anti-aging (Zhou et al., 2001). Both IIS and AMPK pathways converge on mTOR. Nutrient and amino acid starvation inhibits mTOR activity, resulting in suppressed protein translation and enhanced autophagy via key mTOR targets S6K1 (ribosome protein S6 kinase B1), 4EBP1 (eukaryotic translation initiation factor 4E-binding protein 1), and ULK1 (unc-51 like autophagy activating kinase 1) (Wullschleger et al., 2006; Laplante and Sabatini, 2012). Sirtuins are NAD+ (nicotinamide adenine dinucleotide)–dependent deacetylases that act on both histone substrates to silence heterochromatin (Oberdoerffer et al., 2008; Dang et al., 2009) and on non-histone substrates such as P53 (Luo et al., 2001; Vaziri et al., 2001), HSF1 (heat shock factor 1), FOXO (Brunet et al., 2004), and PGC1 (Rodgers et al., 2005), among others, to regulate aging.

Among these regulators, mTOR function has been genetically tackled in several adult stem cell, aging, and malignancy contexts. Hyperactivation of mTOR by deletion of *Pten* or *TSC1* exhausted neural stem cells (Bonaguidi et al., 2011) and HSCs (Yilmaz et al., 2006; Zhang et al., 2006; Gan et al., 2008; Chen et al., 2009), and the HSCs were transformed to become precursors of myeloproliferative disorder. On the other hand, loss of mTOR function via ablation of the mTORC1 component *Raptor* (regulatory-associated protein of mTOR) in mouse HSCs leads to non-lethal pancytopenia, splenomegaly, and accumulation of monocytoid cells. Raptor conditional knockout also compromised HSC regeneration and inhibited leukemogenesis evoked by *Pten* deficiency (Kalaitzidis et al., 2012). p53 and p16 mediate HSC exhaustion and serve as a roadblock to leukemic transformation upon *Pten* deletion (Lee J. Y. et al., 2010b). In adult but not childhood leukemia, deletion of the mTORC2 component *Rictor* blocked leukemogenesis and HSC depletion (Magee et al., 2012). Mechanistically, it has been shown that in *Drosophila* larvae, chronic stimulation of TOR (via constitutively active insulin receptor expression) induces ROS and activates JNK and FOXO, resulting in accumulation of Sestrin (a family of stress-sensing proteins) and activation of AMPK, facilitating autophagic clearance of damaged mitochondria, protein aggregates, or lipids (Lee J. H. et al., 2010a). Loss of Sestrin resulted in age-associated pathologies including triglyceride accumulation, mitochondrial dysfunction, muscle degeneration, and cardiac malfunction, which could be reversed by AMPK activation or TOR inhibition. In the skin, deletion of either *Mtor* itself or its complex components *Raptor* of mTORC1 or *Rictor* of mTORC2 led to skin barrier defects during development (Ding et al., 2016). Treatment with rapamycin (preferentially targeting mTORC1) reversed HFSC exhaustion induced by WNT1 overactivation (Castilho et al., 2009), whereas mTORC2 has been suggested to mediate glutaminase suppression in returning HFSCs to quiescence (Kim et al., 2020).

Calorie restriction or dietary restriction is widely known to ameliorate aging-associated decline of stem cells, such as HSCs (Chen et al., 2003; Warr et al., 2013; Tang et al., 2016), germline stem cells (Mair et al., 2010), and stem cells of the intestine (Regan et al., 2016; Akagi et al., 2018; Mihaylova et al., 2018), skeletal muscle (Cerletti et al., 2012), and skin (Forni et al., 2017; Solanas et al., 2017). Mechanistically, in the context of intestinal stem cells, calorie restriction enhances stem cell activity through a niche-dependent paracrine signal (Yilmaz et al., 2012). In HSCs, calorie restriction cell-autonomously preserves the functional autophagy-high and oxidative phosphorylation–low populations during aging (Warr et al., 2013; Ho et al., 2017). Similar to basal autophagy, chaperon mediated autophagy is also required for maintaining HSC function during aging (Dong et al., 2021). In aged neural stem cells, calorie restriction induced lysosome activation clears aggregates and restores stem cell activation (Leeman et al., 2018), and likewise, chaperon mediated autophagy is essential for preventing proteome collapse and neurodegenerations (Bourdenx et al., 2021). Calorie restriction also restores the transcriptional circadian rhythm of aged epidermal and muscle stem cells to their youthful level, such as replication and autophagy, respectively (Solanas et al., 2017).

By sensing energy demand and nutrient supply, stem cells adaptively tune the level of glycolysis (breakdown of glucose into pyruvate under aerobic conditions or lactate under anaerobic conditions) and oxidative phosphorylation pathways (including oxidation of pyruvate, glutamine, or fatty acid in mitochondria, TCA cycle, electron transport chain activity) (Figure 3B). It is commonly believed that long-term self-renewing stem cells reside in a hypoxic niche and downregulate mitochondrial activity (Mazumdar et al., 2010; Simsek et al., 2010; Takubo et al., 2010). Blocking the influx of glycolytic metabolites into mitochondria by overexpression of pyruvate dehydrogenase kinase (which inhibits pyruvate dehydrogenase and hence pyruvate oxidation) increases the long-term self-renewal capacity of HSCs (Simsek et al., 2010; Takubo et al., 2013), while deletion of mitochondrial pyruvate carrier (which transports pyruvate into mitochondria for oxidation) enhances the organoid-forming potential of intestinal stem cells (Schell et al., 2017). Likewise, in the skin, depletion of mitochondrial pyruvate carrier induced HFSC activation, whereas ablation of lactate dehydrogenase (which converts pyruvate to lactate during anaerobic glycolysis) blocked HFSC activation (Flores et al., 2017). Interestingly, glutamine oxidation is highly upregulated in HFSC progenies compared to HFSCs in organoid culture, and it has been proposed to promote HFSCs returning to quiescence at the end of the hair cycle (Kim et al., 2020). While oxidative phosphorylation and mitochondrial activity are unequivocally induced upon stem cell activation, whether glucose or glutamine influx contributes to the increased oxidation is challenging to directly determine *in vivo*. Tissue-specific depletion of glutaminase (which converts glutamine to glutamate), similar to the experiments performed on mitochondrial pyruvate carrier and lactate dehydrogenase, will likely provide further insights into this interesting question.

Mitochondrial biology has long been intertwined with aging biology (Beckman and Ames, 1998). Mice with mutated mitochondrial DNA polymerase Polg age prematurely (Trifunovic et al., 2004; Kujoth et al., 2005), inducing a differentiation block in tissues with high turnover rate including skin (Norddahl et al., 2011; Ahlqvist et al., 2012). Mice with epidermis-specific loss of TFAM (mitochondrial transcription factor A), required for the transcription of mitochondrial genes encoding electron transport chain subunits, showed impaired epidermal differentiation and hair follicle growth (Hamanaka et al., 2013; Kloepper et al., 2015). Indeed, electron transport chain activity declines in aging, and enhancing mitochondrial function by overexpression of PGC1 (Rera et al., 2011; Dillon et al., 2012) or supply of NAD+ (Gomes et al., 2013) delays aging. Consistently, during aging, telomere dysfunction compromises mitochondrial function *via* p53-mediated inhibition of PGC1 levels (Sahin and Depinho, 2010). In an interesting twist, it has been shown that mild mitochondrial stress prolongs lifespan in worms (Durieux et al., 2011) and yeast (Pan et al., 2011). The specific context under which these pathways operate and the cell types responding to these various manipulations likely play a major role in explaining these seemingly complex observations. It would be of future interest to examine how restoring ETC activity or inducing mild mitochondrial stress would affect skin ageing.

## Deciphering Transcriptional and Epigenetic Noise in Aging Stem Cells

**FIGURE 4:**
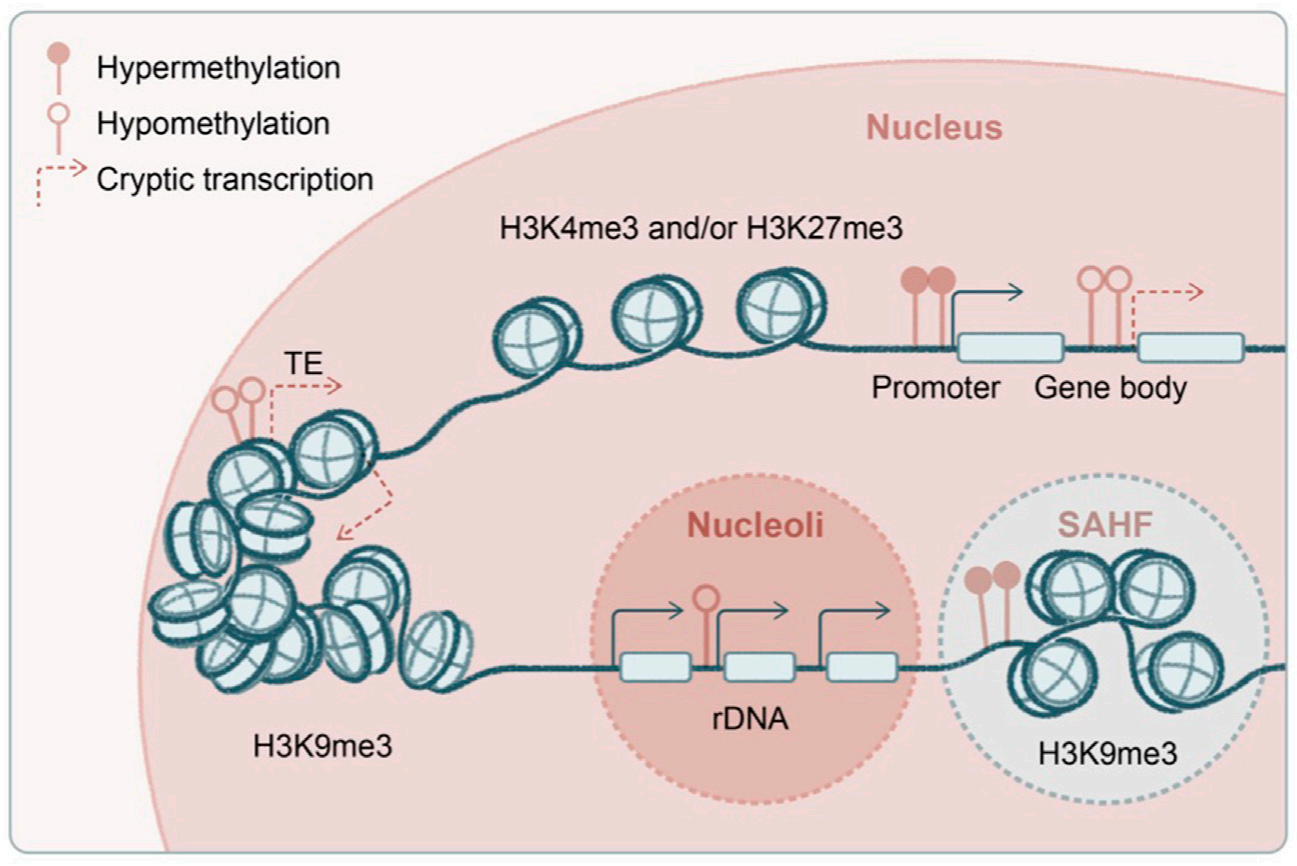
Transcriptional and epigenetic noise in aging stem cells. Several proposed origins of the transcriptional and epigenetic noise in aged stem cells, including genome-wide hypomethylation and derepression of transposable elements, spurious transcription from the gene body, and deregulated transcription from ribosomal DNA (rDNA) at nucleoli. Global hypomethylation is accompanied by regional hypermethylation at gene promoters as well as formation of senescence-associated heterochromatin foci (SAHF). Some of the enzymes and post-translational histone modifications have been shown to regulate aging and longevity in a transgenerational fashion.

A hallmark of aging-associated stem cell degeneration is transcriptional noise and epigenetic erosion, in which heterochromatin silencing and transcriptional fidelity appear crucial. Pioneering genetic and biochemical experiments have mapped out the central pathway for heterochromatin regulation (Jenuwein and Allis, 2001; Zhang and Reinberg, 2001; Grewal and Moazed, 2003; Jaenisch and Bird, 2003; Roeder, 2005; Workman, 2006; Kouzarides, 2007; Zaratiegui et al., 2007; Law and Jacobsen, 2010; Piunti and Shilatifard, 2016; Schuettengruber et al., 2017), a critical vulnerability during organismal aging. Epigenetic deregulations in aging have been extensively characterized in model organisms, where several parallels could be drawn into the mammalian systems including skin (Figure 4).

A key gene-silencing mechanism in mammals, DNA methylation is not conserved or has a very limited role in yeast, flies, and worms. Rather, histone modifications are heavily involved in transcriptional silencing in the latter. For example, during yeast replicative aging, decline of the deacetylase Sir2 results in acetylated histone 4 lysine 16 (H4K16ac) and increased transcription at subtelomeric regions (Dang et al., 2009), as well as enhanced ribosomal DNA (rDNA) transcription by RNA polymerase I and formation of extrachromosomal rDNA circles (Sinclair and Guarente, 1997) (H4K16ac and RNA polymerase I transcription factors are Sir2 substrates). Interestingly, aged HSCs exhibit global DNA hypomethylation that includes the rDNA regions (Sun et al., 2014), and they accumulate non-canonical gH2AX and ATR at nucleoli where rDNA transcription occurs, disrupting ribosome biogenesis (Flach et al., 2014). Notably, SIRT1 (the mammalian homolog of yeast Sir2) appears to contribute to repeat silencing and is involved in the DNA damage response in mammalian cells (Oberdoerffer et al., 2008), although its role in mammalian rDNA regulation is unknown. Since, unlike yeast, mammals use both DNA and histone methylation to mediate transcriptional repression, it will be interesting to see which of these pathways are involved in rDNA silencing during skin aging. Such effort will now be greatly facilitated by the recently completed draft of gapless human genome covering highly repetitive regions including rDNAs (Nurk et al., 2022).

Besides repetitive rDNA, another type of highly abundant repeats in the mammalian genome is transposons (Figure 4). The aging epigenome is shaped by global DNA hypomethylation, typically at gene-poor and late-replicating regions, accompanied by focal hypermethylation at genic regions, bearing strong resemblance to the cancer genome (Cruickshanks et al., 2013; Yuan et al., 2015). In yeast, decline of histone chaperones and global loss of histones during aging lead to transposon derepression and genome instability (Feser et al., 2010; Hu et al., 2014; O'Sullivan et al., 2010). Deficiencies in histone deacetylase SIRT6 in mice (Simon et al., 2019), exonuclease TREX1 in human cells (Thomas et al., 2017), and endonuclease AGO2 in flies (Li et al., 2013) have been shown to induce transposons in aging and inflammatory diseases. In a unique group of premature aging diseases, progeria and laminopathies, lamin-associated domains become disintegrated (Kudlow et al., 2007), accompanied by global loss of H3K9me3 and structural changes in the heterochromatin (Scaffidi and Misteli, 2006; Zhang et al., 2015) and subsequently nuclear autophagy and cytosolic chromatin fragment–induced inflammation (Dou et al., 2015). Indeed, in keratinocytes, it has been shown that mechanical strain leads to H3K9me3 heterochromatin loss at the nuclear lamina and disruption of lineage gene expression during aging (Le et al., 2016; Koester et al., 2021), suggesting a viable hypothesis that deregulation of heterochromatin drives skin aging. Among H3K9me3 methyltransferases, G9a has been functionally examined in the mammalian epithelium. While G9a is minimally involved in homeostasis potentially because of functional redundancy, it is critical for tumorigenesis (Avgustinova et al., 2018; Avgustinova and Benitah, 2021).

Another source of spurious transcription in aging could be emanated from gene bodies (Figure 4). H3K36me3 methyltransferases are among the first discovered epigenetic regulators of longevity: Set2 in yeast (Carrozza et al., 2005; Sen et al., 2015) and Met-1 in worms (Hamilton et al., 2005; Pu et al., 2015). In these model systems, it has been shown that RNA polymerase II–associated methyltransferase deposits H3K36me3 along the gene body, recruiting histone deacetylase to suppress spurious transcription initiated from the gene body, and this transcriptional fidelity becomes compromised in aging. A similar mechanism has recently been described in mammalian cells (Neri et al., 2017; McCauley et al., 2021), in which DNA methyltransferase is likely recruited by H3K36me3 along the gene body to mediate the suppression of cryptic transcription (Dhayalan et al., 2010). The mammalian H3K36me3 is maintained by multiple methyltransferases, Setd2 and Nsd1/2/3, which exhibit both overlapping and distinct functions (Barral et al., 2022).

Both H3K9me3 and H3K36me3 are closely associated with DNA methylation and frequently exhibit interdependency. Epidermis-specific deletion of the *de novo* DNA methyltransferase Dnmt1 led to hair follicle miniaturization along with epidermal and sebaceous gland hyperplasia, a signature of premature aging in the skin (Li et al., 2012). Consistently, in the epidermis, Dnmt1 along with its foreshadowing E3 ubiquitin ligase Uhrf1 are required to maintain stem cell self-renewal (Sen et al., 2010; Mulder et al., 2012), loss of which results in autoinflammatory conditions in the skin (Beck et al., 2021). A related enzyme, Tet2, that catalyzes 5-hydroxymethylcytosine (5hmC) and promotes DNA demethylation, is essential to maintain epidermal differentiation (Boudra et al., 2021). Furthermore, deletion of the maintenance DNA methyltransferases Dnmt3a/b from epithelial cells largely spared skin homeostasis but contributed to malignant transformation (Rinaldi et al., 2016; Rinaldi et al., 2017). Interesting, deletion of mediator complex component Med1 (Nakajima et al., 2013) or the SWI/SNF-like BAF complex catalytic subunit Brg1 (Xiong et al., 2013) also elicits HFSC exhaustion and alopecia phenotype, although in the case of BAF complex, its function is context dependent and regulates either stem cell expansion or differentiation based on which lineage is examined (Bao et al., 2013; Mardaryev et al., 2014).

Another repressive histone post-translational modification proposed to regulate aging is the inactive or poised chromatin marker H3K27me3, which declines due to increased demethylase UTX-1 during aging (Jin et al., 2011; Maures et al., 2011). In this case, the impact of H3K27me3 loss on aging has been attributed to its specific regulation of the IIS (insulin/insulin-like growth factor) genes. In contrast, the excessive levels of the active chromatin marker H3K4me3 and its responsible methyltransferase, trithorax, has been shown to be detrimental to longevity (Greer et al., 2010; Greer et al., 2011). On the other hand, H3K4me3 demethylase promotes longevity in worms, with a remarkable transgenerational memory effect observed in this context (Greer et al., 2014). Mild mitochondrial stress-induced lifespan extension is associated with epigenetic remodeling via histone methyltransferases (Tian et al., 2016) and demethylases (Merkwirth et al., 2016). Remarkably, genome-wide mapping of 5mC and 5hmC in HSCs revealed extended regions of low-methylation canyons that are distinct from CpG islands and shores (Jeong et al., 2014). These canyons harbor either H3K27me3 or poised H3K27me3/H3K4me3 histone markers, whose borders are demarcated by 5hmC, and are deregulated upon Dnmt3a/Tet1 deletion or in aging (Jeong et al., 2014; Sun et al., 2014; Dixon et al., 2021).

In skin, histone methyltransferases of H3K27me3 (polycomb group) and H3K4me3 (trithorax group) regulate epidermal stem cell self-renewal and differentiation, respectively, during development and regeneration (Millar, 2011; Frye and Benitah, 2012; Perdigoto et al., 2014; Guan et al., 2020). For example, in PRC2 Ezh2 conditional knockout, P16 is derepressed, resulting in cell cycle arrest and epithelium hypoplasia (Ezhkova et al., 2009), similar to PRC1 deficiency (Cordisco et al., 2010; Luis et al., 2011). In contrast, the H3K27me3 demethylase promotes epidermal differentiation (Sen et al., 2008). Likewise, histone deacetylases HDAC1/2 (LeBoeuf et al., 2010) (Hughes et al., 2014) and HDAC3 (Szigety et al., 2020) have opposing functions in stem cell proliferation versus differentiation. On the other hand, the histone methyltransferase MLL4 (KMT2D) that catalyzes H3K4me1 is required to maintain epidermal homeostasis, whose loss resulted in the disruption of skin stratification and lipid metabolism (Lin-Shiao et al., 2018; Egolf et al., 2021). Interestingly, lack of Bmi1, a component of PRC1, leads to impaired mitochondrial function and to increased ROS and DNA damage, which could be reversed by antioxidant treatment or genetic disruption of Chk2 (Liu et al., 2009). These findings suggest that global chromatin disruption may override defects from specific gene deregulations, that it is not an individual gene or a few genes per se that elicit the phenotype. These molecular and cellular mechanisms are potentially extendable to aging.

Compared to global hypomethylation, equally noteworthy is the accompanying focal hypermethylation in aging chromatin (Figure 4). A prominent feature in cellular senescence is the formation of the senescence-associated secretory phenotype (SASP) on one hand (Coppé et al., 2010) and senescence-associated heterochromatin foci (SAHF) on the other (Narita et al., 2003; Zhang et al., 2005; Di Micco et al., 2011; Rai et al., 2014; Ricketts et al., 2015). Remarkably, a sparse hair coat in aged mice is recovered upon FOXO4 peptide treatment, which blocks p53 nuclear localization and induces the apoptosis of senescent cells (Baar et al., 2017), supporting the functional significance of senescence in skin aging. SAHF has been suggested as a survival mechanism to “plug the hole” that assembles heterochromatin onto euchromatin regions, given the compromised nuclear lamina that otherwise is necessary to maintain the physiological heterochromatin. Alternatively, one could speculate these foci may titrate away rate-limiting factors (H3.3, methyltransferase, or metabolites required for transcriptional repression; see Discussion) from transposon repeats and heterochromatic regions, exacerbating the already-weakened epigenetic regulation during aging. Although this model has not been formally tested in aging, a similar titration- or competition-based inhibitory mechanism from H3K27M and H3K36M oncohistones has been shown in cancer (Lewis et al., 2013; Lu et al., 2016), leaving the field with the intriguing question of how the lineage specificity and gene selectivity is achieved in the context of global epigenetic deregulations.

## Discussion

While the identity of upstream signals that lead to defective epigenetic machinery in aging remains unclear, it appears safe to assume these factors would be rate-limiting. As mentioned, nutrients and metabolites, in addition to their impact on cell signaling transductions and mitochondrial biology, are appealing candidates that appear to dictate the aging transcriptome and epigenome. Many epigenetic enzymes that regulate methylation or acetylation exhibit K_M_ in the range of observed substrate concentrations in cells, suggesting rates of these reactions are highly sensitive to the cellular fluctuations of the corresponding metabolites, such as S-adenosyl methionine, acetyl-CoA, alpha-ketoglutarate, NAD+, and beta-hydroxybutyrate (Schvartzman et al., 2018), raising the intriguing question of the extent to which cellular metabolite deregulation shapes the aging epigenome. Indeed, it has been shown that in autophagy-deficient HSCs, DNA methylation and lineage gene expression are regulated by S-adenosyl methionine and alpha-ketoglutarate levels (Ho et al., 2017). Ascorbate and its downstream target TET2 dictate HSCs (Agathocleous et al., 2017) as well as (Boudra et al., 2021) epidermal function and tumorigenesis. NAD+ being the rate-limiting factor, competition between PARP-1 (also NAD+ dependent) and SIRT1 dictates cellular response in DNA damage and epigenome regulation (Bai et al., 2011) (Scheibye-Knudsen et al., 2014). RNA sequencing (RNA-seq) and assay for transposase-accessible chromatin with sequencing (ATAC-seq) at single cell level, and together with spatial transcriptomics provided unprecedented throughput and resolution of cell type, lineage trajectory, and tissue-level crosstalk in many biological contexts, and are likely to be powerful technologies in aging research. The current challenge lies in the metabolomics at a sub-cellular level and within the intact organism *in vivo*, both of which are critical to understand molecular determinants of aging.

Local tissue microenvironment plays a dominant role in dictating stem cell function, including aging. Aged dermal fibroblasts maintain their positional identity (Marsh et al., 2018) while manifesting adipogenic and inflammatory traits (Salzer et al., 2018; Mahmoudi et al., 2019). Secreted factors of BMP and WNT pathways enriched in adipocytes (Keyes et al., 2013; Chen et al., 2014) and pre-adipocytes (Festa et al., 2011) known to dictate hair follicle regeneration are disrupted in aged dermis (Chen et al., 2014; Chen et al., 2015). Skin-resident immune cells are drastically remodeled during aging (Giangreco et al., 2008; Doles et al., 2012), including regulatory T cells that are known to support hair follicle regeneration (Ali et al., 2017) and decline significantly in aged dermis (Ge et al., 2020). So are the arrector pili muscles and nerves (Brownell et al., 2011; Fujiwara et al., 2011; Shwartz et al., 2020) that provide niche input to HFSCs, both of which become dislodged in aged skin (Ge et al., 2020). Of significance, aged HFSCs can be rejuvenated by resident cell types and niche components of the young skin (Chen et al., 2014; Ge et al., 2020; Koester et al., 2021), suggesting that the tissue microenvironment drives stem cell function during aging. Future work will need to exploit the molecular identity, regulatory signals, and therapeutic potential of such rejuvenation factors.
